# Neuroendocrine characteristics of induced pluripotent stem cells from polycystic ovary syndrome women

**DOI:** 10.1007/s13238-018-0600-1

**Published:** 2018-12-10

**Authors:** Zheying Min, Yue Zhao, Jing Hang, Yun Ren, Tao Tan, Yong Fan, Yang Yu

**Affiliations:** 1 Key Laboratory for Major Obstetric Diseases of Guangdong Province, The Third Affiliated Hospital of Guangzhou Medical University, Guangzhou, 510150 China; 2 Beijing Key Laboratory of Reproductive Endocrinology and Assisted Reproductive Technology and Key Laboratory of Assisted Reproduction, Ministry of Education, Center for Reproductive Medicine, Department of Obstetrics and Gynecology, Peking University Third Hospital, Beijing, 100191 China; 3 Peking-Tsinghua Center for Life Sciences, Academy for Advanced Interdisciplinery Studies, Peking University, Beijing, 100871 China; 4 Yunnan Key Laboratory of Primate Biomedical Research, Institute of Primate Translational Medicine, Kunming University of Science and Technology, Kunming, 650500 China

**Dear Editor,**

Polycystic ovary syndrome (PCOS) is a common female reproductive endocrinopathy that afflicts up to 10%–15% of women in reproductive age worldwide (Nestler, 2016). Women with PCOS exhibit hyperandrogenism, intermittent/absent menstrual cycles, and polycystic ovaries on ultrasound (Rotterdam, 2004). The pathophysiology of PCOS extends beyond infertility and hirsutism to hypothalamic neuroendocrine dysfunction (Goodarzi et al., 2011). Most women with PCOS exhibit increased luteinizing hormone (LH) levels, resulting from high-frequency gonadotropin-releasing hormone (GnRH) secretion (Cimino et al., 2016). Prenatal testosterone (T) treatment in sheep results in disrupted steroid feedback on gonadotropin release, which increases pituitary sensitivity to GnRH and subsequently leads to LH hypersecretion (Sullivan and Moenter, 2004; Cardoso et al., 2016). A recent study shows that GnRH-dependent LH pulsatility and secretion are elevated by anti-Müllerian hormone (AMH) in PCOS disease. The increased prenatal AMH reprograms fetus and induces PCOS in adults (Tata et al., 2018). Furthermore, the androgen receptor (AR) plays a role in hyperandrogenism and ovarian folliculogenesis in PCOS (Wang et al., 2015; Abbott, 2017). However, the disease mechanism behind PCOS remains unclear, and current management focuses on treating the symptoms but not the mechanism (Chen et al., 2016; Shi et al., 2018). A further understanding of this disease is necessary to uncover the pathology of PCOS and develop new potential therapeutic avenues and drugs.

To establish a cell model for investigating PCOS disease, we first generated iPSCs from the skin tissues of three PCOS- and three non-PCOS patients. The primary fibroblasts of the patients were transduced with lenti-viral vectors expressing OCT4, SOX2, C-MYC and KLF4 according to a direct reprogramming protocol (Okita et al., 2011) (Fig. S1A). The generated iPSCs displayed typical pluripotent stem cell morphology (Fig. 1B), expressed pluripotency markers of OCT4, NANOG and SOX2 (Fig. 1C), and displayed normal karyotypes (Fig. S1B). These results showed successful derivation of iPSCs from patients.

**Figure 1:**
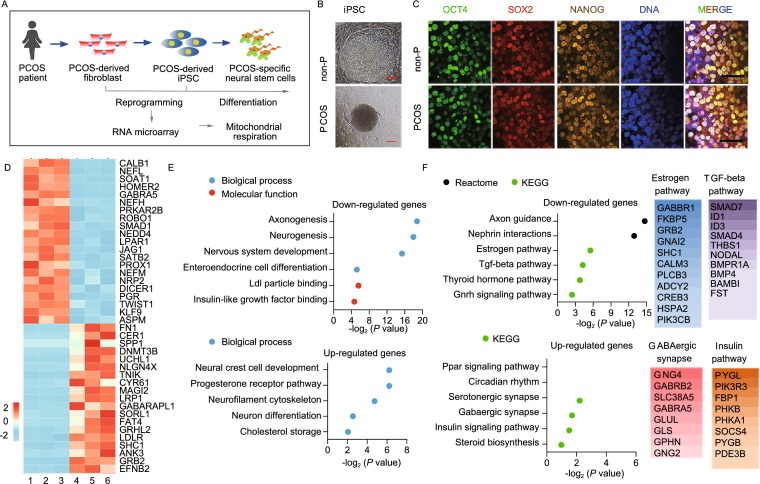

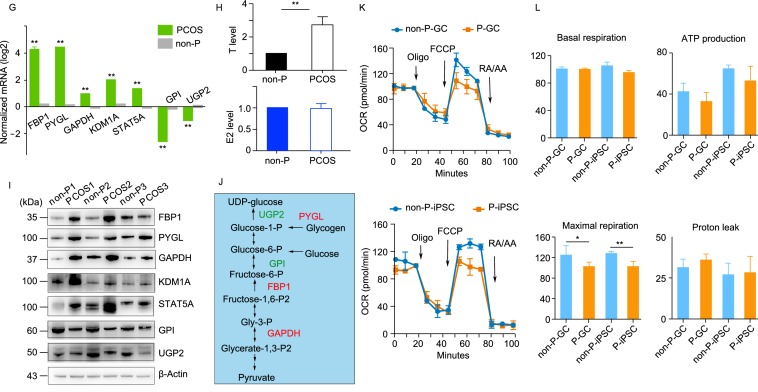
**The metabolic and neuroendocrine characteristics of PCOS-derived iPSCs**. (A) A proposed schedule of establishing a PCOS cell model using iPSCs, microarrays and cell differentiation methods. (B) The phenotype of PCOS-derived iPSCs from patients. Scale bars = 100 µm. non-P: non-PCOS. (C) Immunofluorescence images of pluripotent markers OCT4, SOX2 and NANOG of iPSCs. Scale bars = 50 µm. (D) Top 40 neuroendocrine-related genes shown in a heatmap (*n* = 3). (E) The down-regulated genes and up-regulated genes in GO terms based on molecular function and biological process. (F) The down-regulated and up-regulated genes annotated with KEGG and the Reactome database. (G) RT-qPCR verification of up- and down-regulated genes in the microarray. The error bars represent ±SD; **P < 0.01. (H) ELISA verification of the testosterone (T) and estradiol (E2) expression levels of PCOS-derived iPSC cultured medium (*n* = 3). (I) Western blot analysis of DEGs corresponding to RT-qPCR. 1–3: PCOS-1 to PCOS-3; 4–6: non-PCOS-1 to non-PCOS-3. (J) Diagram of glycolysis pathway. Red: increased genes in PCOS; Green: decreased genes in PCOS. (K) The mitochondrial respiration ability of PCOS GCs and PCOS-derived iPSCs in response to oligomycin, carbonyl cyanide 4-(trifluoromethoxy) phenylhydrazone (FCCP), rotenone and antimycin (RA/AA). (L) Quantitative analysis of basal oxygen consumption, ATP production, maximal respiration and proton leak

Next, the total RNA was extracted from PCOS- and non-PCOS-derived iPSCs for RNA microarray analysis. The global transcriptional genes of PCOS-derived iPSCs were identified and enriched by gene ontology (GO). Filtering by *P* value less than 0.01 and fold-changes of more than 2, 2,904 differentially expressed genes (DEGs) were collected between PCOS- and non-PCOS-derived iPSCs. Among these DEGs, we were interested in genes related to neuroendocrine and metabolic processes in PCOS. The enriched top 40 up- and down-regulated genes were shown in heatmaps (Fig. 1D). The GO enrichment revealed that down-regulated transcripts were associated with neurogenesis, enteroendocrine cell differentiation and LDL particle binding process. Up-regulated transcripts were enriched in neural crest cell development, progesterone receptor pathway and cholesterol storage process (Fig. 1E). We focused on genes of GABA receptor, CYP family, TGF-β pathway and estrogen receptor pathway in neuroendocrine processes (Fig. 1F). Then seven significantly modulated genes (*FBP1*, *PYGL*, *GAPDH*, *KDM1A*, *STAT5*, *GPI* and *UGP2*) were verified by RT-qPCR, according to functional annotation and their fold change (Fig. 1G). Moreover, the protein levels of above genes were verified by Western blot in PCOS-derived iPSCs, demonstrating the similar changes with mRNA levels (Fig. 1H). These genes (*FBP1*, *PYGL*, GAPDH, *GPI* and *UGP2*) involved in glycolysis process were expressed abnormally, indicating deregulation of glucose metabolism (glycolysis and gluconeogenesis) in PCOS (Fig. 1J). In addition, we measured the expression levels of testosterone (T) and estradiol (E2) in the cultures of iPSCs via ELISA (enzyme-linked immunosorbent assay), to verify the neuroendocrine characteristics in PCOS. The results showed that PCOS-derived iPSCs secreted greater T significantly than non-PCOS-derived iPSCs, while there were no significant differences in E2 level between PCOS and non-PCOS-derived iPSCs (Fig. 1H). The change of T level was consistent with the clinical hyperandrogenic feature of PCOS (Haouzi et al., 2012). In order to compare the differences of metabolic function after reprogramming, we measured the mitochondrial respiration ability of ovarian granulosa cells (GCs) and iPSCs from PCOS and non-PCOS patients. The GCs were obtained from other three PCOS and non-PCOS patients. The results showed that the maximal respiration ability of GCs and iPSCs from PCOS patients were decreased (Fig. 1K and 1L), which suggested the metabolic ability of PCOS was deregulated. The attenuated mitochondrial ability corresponded to the deregulated endocrine and metabolism genes of functional annotation.

Finally, for better understanding the biological effects of deregulated neuroendocrine genes in PCOS, the neural stem cells (NSCs) were differentiated from PCOS- and non-PCOS-derived iPSCs according to a retinoic acid (RA) induction method (Zhang et al., 2018) (Fig. 2A). Briefly, PCOS- and non-PCOS-derived iPSCs were suspended in culture medium for 4 days to induce embryoid bodies (EBs). iPSCs were supplemented with RA and cultured for 4 days for NSC differentiation, and then the RA was removed. On day 12, most of these cells displayed NSC morphology (Fig. 2B). The neural progenitor markers of NESTIN and SOX2 were identified via immunofluorescence staining (Fig. 2C). Finally, we measured the mitochondrial respiration ability of NSCs from PCOS- and non-PCOS patients. The NSCs of PCOS displayed similar decreased maximal respiration ability, compared to that of PCOS-derived iPSCs (Fig. 2D and 2E).

**Figure 2:**
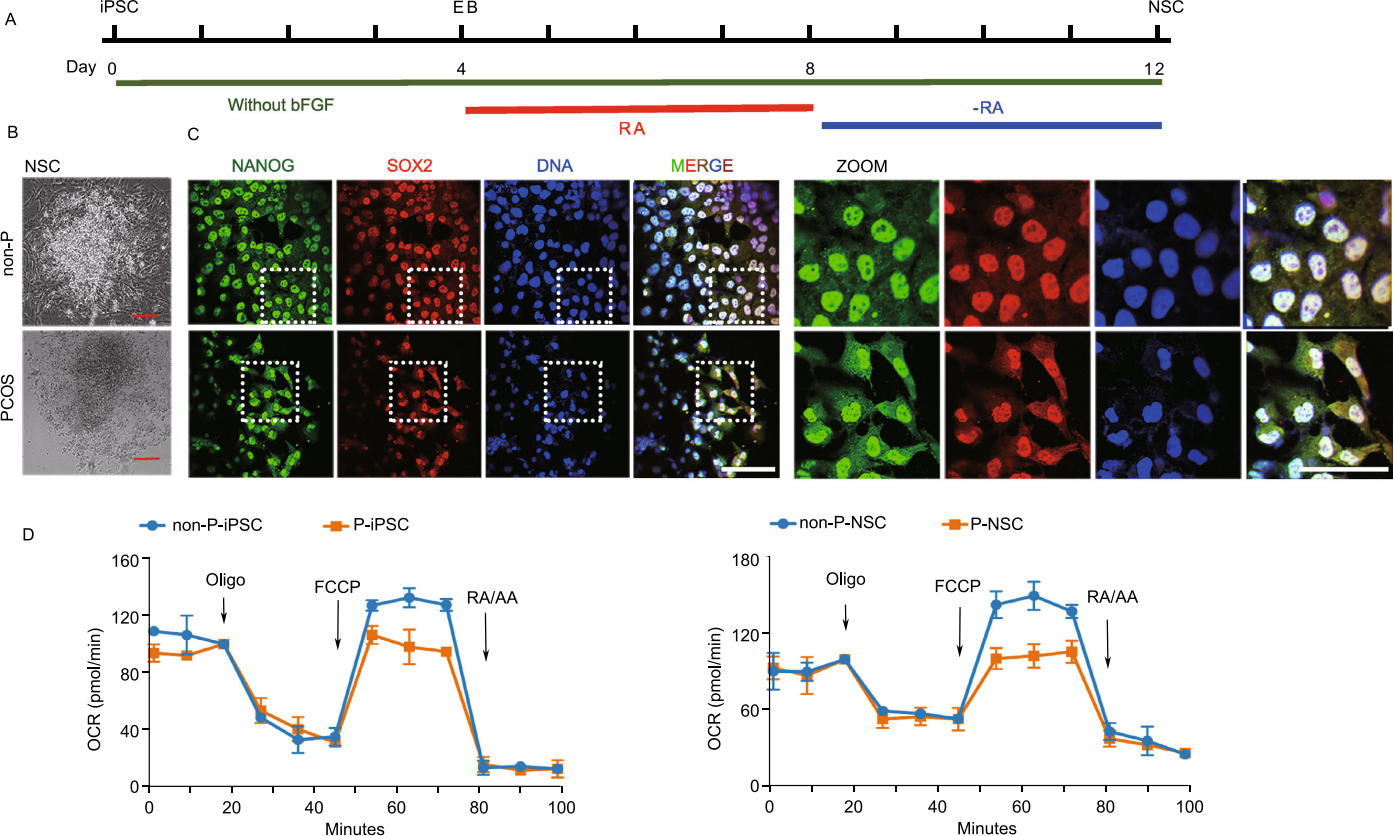

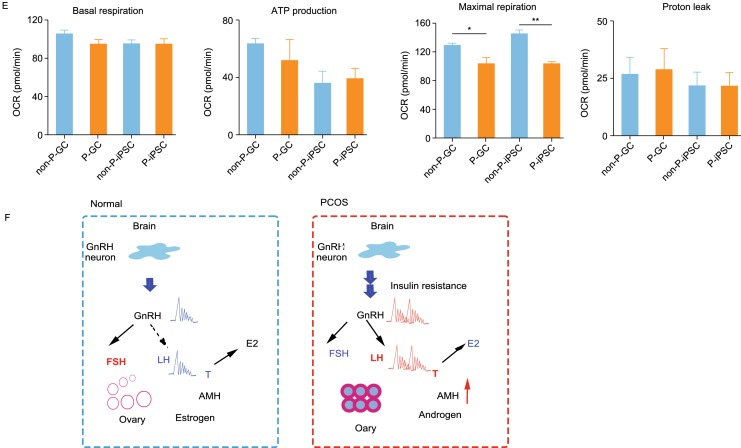
**Differentiation and identification of NSCs from PCOS-derived iPSCs**. (A) Schematic procedure of NSCs differentiation from iPSCs. NSC: neural stem cell; EB: embryoid body. (B) The phenotype of specific differentiated NSCs. Scale bars = 100 µm. (C) Immunofluorescence images of the NSC markers SOX2 and NESTIN. Scale bars = 50 µm. ZOOM, scale bars = 25 μm. (D) The mitochondrial respiration functions of PCOS- and non-PCOS-derived iPSCs and NSCs. (E) Quantitative analysis of basal oxygen consumption, ATP production, maximal respiration and proton leak. (F) Proposed neuroendocrine state in normal and PCOS patients. In normal patients, the GnRH pulsatile frequency is critical for steroidogenesis and follicular development. Low frequency pulses prefer FSH, and high frequency pulses favour LH. In PCOS, the increased GnRH release led to a high level of LH pulsatility, impairing the preferential release of FSH and follicular maturation, thus leading to polycystic ovaries. Red: increased; Blue: decreased. Solid arrow: up regulated; Dotted arrow: down regulated.

PCOS is a complicated and multifactorial metabolic syndrome. Elevated androgen levels, disturbed menstrual cycles and increased LH are the main features of PCOS disease. The accurate regulation of the hypothalamic-pituitary-gonadal axis involves some intrinsic factors including estrogen, progesterone, activin and inhibin, and extrinsic factors including neurotransmitters and stress. Any interference with or deregulation of these factors may result in reproductive endocrine irregularity. GnRH neurons play a role in the central regulation of fertility. A pulsatile signal of GnRH is necessary for the secretion of LH and FSH (follicle-stimulating hormone), which promotes steroidogenesis and follicular development in females (Chaudhari et al., 2018). In the altered hypothalamic-pituitary-gonadal axis, high levels of LH reflect increased GnRH release. GnRH receives input from GABAergic neurons, and GABA type A receptor activation may play a part in regulating steroids (Sullivan and Moenter, 2004) (Fig. 2F). Those deregulated genes enriched in glucose metabolic process, demonstrated depressed glycolysis process, decreased glucose transport and increased gluconeogenesis, which can attenuate metabolic ability and lead to glucose intolerance and insulin resistance in PCOS. Moreover, the differentially expressed genes were partially consistent with previously reported microarray profiles of cumulus cells of PCOS patients (Haouzi et al., 2012). However,the clinical cause and molecular mechanism of PCOS is still unclear. PCOS is considered a heredity disease with interaction of genomic variants and environmental factors. Three patients in each group were not enough to generalize all types of PCOS, although three iPSC lines showed the consistent change trends. Furthermore, it is necessary to be replicated and verified in other cohorts and clinical samples.

In this study, we identified neuroendocrine and metabolic characteristics of iPSCs from women with PCOS and non-PCOS. GO analysis on transcripts of PCOS-derived iPSCs reflected the neuroendocrine abnormalities of PCOS, and the mitochondrial respiration ability unfolded metabolic weaknesses in PCOS disease. The NSCs differentiated from PCOS-derived iPSCs showed decreased mitochondrial respiratory ability, similar to iPSCs and primary GCs from PCOS patients. This study provided a cell model demonstrating PCOS characteristics *in vitro*, which can be used to further understand the pathology and develop new potential therapeutic avenues of PCOS.

## Electronic supplementary material

Below is the link to the electronic supplementary material.

Supplementary material 1 (PDF 410 kb)
