# Correction to: Neuroendocrine characteristics of induced pluripotent stem cells from polycystic ovary syndrome women

**DOI:** 10.1007/s13238-019-00664-y

**Published:** 2019-11-04

**Authors:** Zheying Min, Yue Zhao, Jing Hang, Yun Ren, Tao Tan, Yong Fan, Yang Yu

**Affiliations:** 1 Key Laboratory for Major Obstetric Diseases of Guangdong Province, The Third Affiliated Hospital of Guangzhou Medical University, Guangzhou, 510150 China; 2 Beijing Key Laboratory of Reproductive Endocrinology and Assisted Reproductive Technology and Key Laboratory of Assisted Reproduction, Ministry of Education, Center for Reproductive Medicine, Department of Obstetrics and Gynecology, Peking University Third Hospital, Beijing, 100191 China; 3 Peking-Tsinghua Center for Life Sciences, Academy for Advanced Interdisciplinery Studies, Peking University, Beijing, 100871 China; 4 Yunnan Key Laboratory of Primate Biomedical Research, Institute of Primate Translational Medicine, Kunming University of Science and Technology, Kunming, 650500 China

## Correction to: Protein Cell 2019, 10(7):526–532 10.1007/s13238-018-0600-1

In the original publication the Fig. 2 and the Supplementary Material 1 was incorrect. The correct version of Fig. 2 and the Supplementary Material are provided in this correction article.

**Figure 2:**
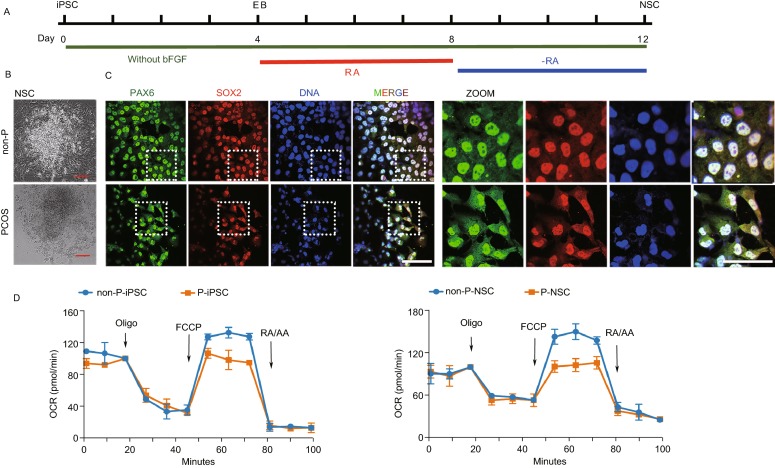

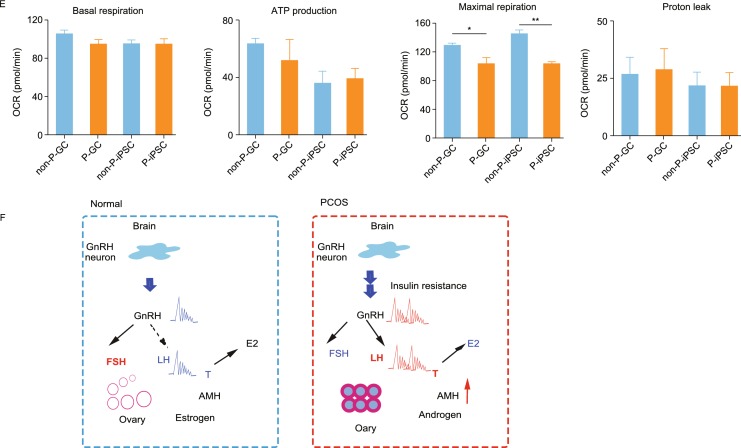
Differentiation and identification of NSCs from PCOS-derived iPSCs. (A) Schematic procedure of NSCs differentiation from iPSCs. NSC: Neural stem cell; EB: embryoid body. (B) The phenotype of specific differentiated NSCs. Scale bars = 100 µm. (C) Immunofluorescence images of the NSC markers SOX2 and PAX6. Scale bars = 50 µm. ZOOM, scale bars = 25 μm. (D) The mitochondrial respiration function of PCOS- and non-PCOS-derived iPSCs and NSCs. (E) Quantitative analysis of basal oxygen consumption, ATP production, maximal respiration, and proton leak. (F) Proposed neuroendocrine state in normal and PCOS patients. In normal patients, the GnRH pulsatile frequency is critical for steroidogenesis and follicular development. Low frequency pulses prefer FSH, and high frequency pulses favour LH. In PCOS, the increased GnRH release led to a high level of LH pulsatility, impairing the preferential release of FSH and follicular maturation, thus leading to polycystic ovaries. Red: increased; Blue: decreased. Solid arrow: up regulated; Dotted arrow: down regulated

NESTIN should be corrected to PAX6 in Fig. 2C legend and at page 528 and Supplementary Material 1. NANOG should be corrected to PAX6 in Fig. 2C picture.

## Electronic supplementary material

Below is the link to the electronic supplementary material.

Supplementary material 1 (PDF 320 kb)

