# Supplementary material for: Correction to: Neuroendocrine characteristics of induced pluripotent stem cells from polycystic ovary syndrome women
Source: Protein Cell. 2019 Nov 4;11(3):227–9. doi: 10.1007/s13238-019-00664-y (PMC7026241; doi:10.1007/s13238-019-00664-y)
Supplement: Supplementary file 1 — Supplementary material 1 (PDF 320 kb) [file 13238_2019_664_MOESM1_ESM.pdf]

Supplemental Information

Materials and Methods

Diagnostic criterion of PCOS patients

The study was approved by Institutional Review Board and Ethics Committee of Peking University Third Hospital. Three PCOS patient and three non-PCOS patient were selected. In this study, subjects who had two of the following three items were diagnosed with PCOS： (1) Oligo or anovulation; (2) Hyperandrogenism; (3) Polycystic ovaries on ultrasonography (Norman et al., 2007).

Generation and culture of PCOS-derived iPSCs

Human fibroblasts were derived from skin cells of patients. The iPSC clones were reprogrammed as described previously. Briefly, epithelial cells were transduced with *OCT4*-, *SOX2*-, *KLF4*- and *C-MYC*-expressing lentiviral vectors in MEF (mitomycin-C-treated mouse embryonic fibroblasts) medium without serum. On day 5, the medium was replaced with iPSC medium [DMEM/F12 supplemented with 20% (v/v) knock out serum replacer (Knockout SR), 2 mM L-glutamine, 2 mM non-essential amino acids, and 0.1 mM β-mercaptoethanol (Invitrogen) with no additional bFGF]. Putative PCOS-derived iPSC colonies were emerged within 21 days after transduction. Generated colonies were mechanically dissociated for passage.

Neural stem cell differentiation from iPSCs

NSC differentiation was performed as described previously. iPSC were picked from

the MEF feeder and suspended cultured for 4 days in iPSC medium without bFGF, to induce embryoid bodies (EB). For NSC differentiation, RA (Retinoic acid) was added at final concentration of 1-2 μM after EB formation. After 4 days as a floating culture, EBs were collected and plated onto matrigel coated dishes cultured in EB medium without RA.

Microarray analysis

Microarray hybridization was carried out at CapitalBio (Beijing, China). Total RNA (100 ng) was used to prepare twice-amplified and labelled RNA for hybridization with HG-UI33 plus 2.0 arrays (n=3).

Quantitative real-time PCR

According to the manufacturer's protocol, total RNA was extracted using Trizol reagent, and cDNA was synthesized by a ReverAid First Strand cDNA Synthesis Kit (Invitrogen). The PCR products were amplified using the SYBR Green mix kit by an QuantStudio3 system (Applied Biosystems). Fold-change by RT-PCR was measured and calculated by normalizing to the housekeeping gene (β-actin) and calculating the average comparative threshold cycle (ΔC_t). Statistical analyses were conducted by *t*-test. RT-PCR Primers used in study are designed in Primer 3.0. Primers used are listed as follows:

FBP1-F, 5'-CTGCCGTCACTGAGTACATC-3';

FBP1-R, 5'-ATATCCCTCCGTAGACCAGAG-3';

PYGL-F, 5'-GAAGAAGATGCTGGACTTGGC-3';

PYGL-R, 5’-TTCTACCTGCCATCCATCTCG-3’;

GAPDH-F, 5’-ACATCATCCCTGCCTCTACTG-3’;

GAPDH-R, 5’-CCTGCTTCACCACCTTCTTGA-3’;

LSD1-F, 5’-ACCTCTCTCAACTCTCTCCCT-3’;

LSD1-R, 5’-GCGGGTATTCACAGCTATCAC-3’;

STAT5-F, 5’-GGTGAGATCCTGAACAACTGC-3’;

STAT5-R, 5’-CTGCCAACACTGAACTGAGAC-3’;

GPI-F, 5’-CTGCCCTATGACCAGTACCTG-3’;

GPI-R, 5’-CACAGGGTATCATCTTGGTGC-3’;

UGP2-F, 5’-GAAGGCAAACTGAGACTGGTG-3’;

UGP2-R, 5’-CATTCAGGCCTCCATCCAAAG-3’.

Immunofluorescence staining

Cells were fixed in 4% (w/v) paraformaldehyde in phosphate-buffered saline (PBS) for 20 min and then blocked with PBS containing Triton X-100 (Sigma-Aldrich) for 30 min at room temperature. After blocking, the cells were incubated with primary antibodies overnight at 4 ℃, then incubated with secondary antibodies 1hr at room temperature. The primary antibodies (all at a 1:250 dilution, Abcam) were used to detect OCT4, SOX2, and NANOG expression of iPSC. NSC was stained by primary antibody of SOX2 and PAX6 (proteintech). We visualized antigen localization using goat anti-mouse/rabbit Alexa Fluor 488, 555 and 594. The nuclei were stained with Hoechst (Invitrogen).

Western blotting

According to the western blotting protocol, iPSCs were collected and homogenized by RIPA buffer. Antibodies: FBP1, GPI, PYGL were diluted with 1:2000 (proteintech), GAPDH and UGP2 were diluted with 1:2000 (abcam), KDM1A and STAT5A were diluted with 1:1000 (CST).

ELISA

The culture medium of iPSC were collected and centrifuged with 12,000 rpm in 4 ℃ before assays. The testosterone and estrodiol levels were detected using ELISA kit (R&D system).

Mitochondrial oxygen consumption detection

The mitochondrial respiration was detected using the XF24 extracellular flux analyzer from Seahorse Bioscience (Billerica). A classical Mito stress test was performed based on the following procedure: (1) the basal respiration was measured before adding chemicals; (2) oligomycin (2.0 μM) was added to inhibit ATP production; (3) The maximal respiration was measured by adding the uncoupler carbonyl cyanide 4-(trifluoromethoxy) phenylhydrazone (FCCP); and (4) rotenone and antimycin A (0.5 μM) were applied in combination to block respiration. The final results were normalized to cell number (10^5 cells per well).

Statistical analysis

The microarray data were analysed to identify statistically significantly different expression. The list of identified genes (fold change, FC>2; FDR<0.05) was submitted to AmiGO2 and DAVID database to identify the Gene Ontology (GO)

terms associated with biological functions and pathways. Data are presented as the mean ±SD and were analysed using GraphPad Prism 5 program (GraphPad Software) and R language package.

Reference:

Norman, R.J., Dewailly, D., Legro, R.S., and Hickey, T.E. (2007). Polycystic ovary syndrome. Lancet 370, 685-697.

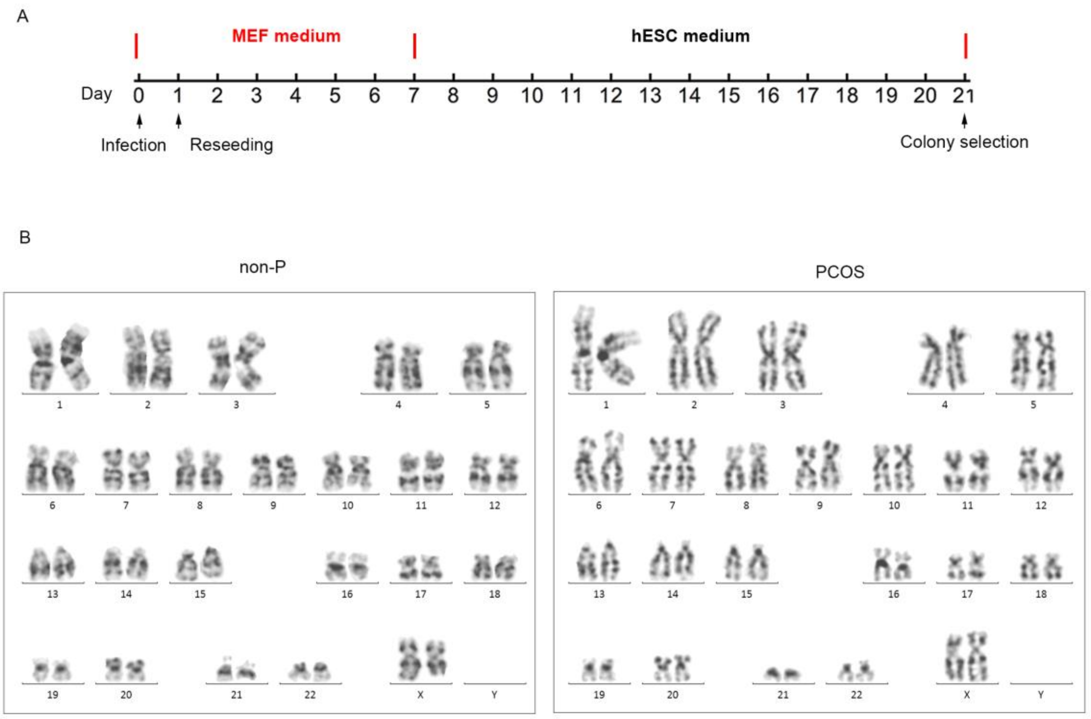

Figure S1. The establishment of PCOS- and non-PCOS-derived iPSCs.

(A) Time line of PCOS-derived iPSC generation from patients.

(B) Karyotype of PCOS- and non-PCOS-derived iPSCs.
